# From past to future: bibliometric perspective of international research activity on lateral epicondylitis

**DOI:** 10.3389/fsurg.2025.1529142

**Published:** 2025-05-20

**Authors:** Boshen Shu, Shufeng Zhang, Jian Gao, Lin Wang, Xiaohui Wang

**Affiliations:** Department of Pediatric Surgery, Henan Provincial People’s Hospital, Zhengzhou, Henan, China

**Keywords:** humeral epicondylitis, tennis elbow, citations, visualized analysis, impact factor

## Abstract

**Introduction:**

Lateral epicondylitis, also termed as “tennis elbow”, is the most common reason for elbow pain and dysfunction. This study aimed to assess the research activity on lateral epicondylitis worldwide.

**Methods:**

Publications on lateral epicondylitis from Web of Science database were recorded and analyzed in June 2024. For each article, citations, authors, title, organization, country, journal, year, keywords, topic, and H-index were extracted. VOSviewer and Excel 2020 were used to operate the bibliometric and visualized study.

**Results:**

A total of 913 publications between 1950 and 2023 were included. The total number of citations was 27,866 with the average citation per publication of 31 times. “Orthopedics” became the dominant topic (*n* = 365, 40.0%). The United States contributed the most publications (*n* = 201, 22.0%). The latest keywords “platelet rich plasma”, “autologous conditioned plasma”, and “extracorporeal shockwave therapy” mainly appeared since 2018.

**Conclusion:**

This bibliometric study indicates that there is a growing trend in the number of publications on lateral epicondylitis. The United States dominated studies of lateral epicondylitis and the *Journal of Shoulder and Elbow Surgery* was the most productive journal. “Platelet rich plasma”, “autologous conditioned plasma”, and “extracorporeal shockwave therapy” may become new interests in lateral epicondylitis research.

## 1 Introduction

Lateral epicondylitis (LE), also termed as “tennis elbow”, is the most common reason for elbow pain and dysfunction, which was initially described by Runge in 1873, but the name derived from “Lawn Tennis Arm” described by Morris in 1883 (1–3). The incidence of LE in adults has been reported between 1% and 3% without gender difference, which is the most prevalent in the fifth decade of life (3, 4). A huge social and economic burden can be caused by LE due to the loss of workdays (5, 6).

Bibliometric analysis, based on the characteristics of publications, performs as a statistical approach to quantitatively and qualitatively analyze the scientific impact, cooperation network and publication trends to grab the spotlight of research (7). VOSviewer is a software on the basis of the Java environment to construct and visualize bibliometric networks for publications. It provides a visual analysis of scientific works through novel insights such as clustering, superposition, and density, which can indicate the present research status, hot topics, and trends of a special field from multiple perspectives (8). This method has been widely used in various research fields (9–11). To date, several bibliometric studies have been conducted on LE (12, 13). However, these studies limited the range for research, only focused on highly cited articles or a specific time span, limiting its' usefulness to the field. Yet, investigating bibliometric characteristics of publications on LE via a more comprehensive way would provide more information in the clinical and basic science research in this domain. We aimed to use VOSviewer as a core tool to analyze all publications related to LE for getting a thorough understanding of current research status and future hotspots.

## 2 Materials and methods

### 2.1 Data source and searching strategy

All accessible peer-reviewed scientific publications on LE were identified via the Web of Science Core Collection (WOSCC) database. It includes Science Citation Index Expanded, Emerging Sources Citation Index, Social Sciences Citation Index, Book Citation Index-Science, and Conference Proceedings Citation Index-Science. The retrieval period was between 1945 and 2023, and the final retrieval execution time was on June 10, 2024. In our study, to analyze only relevant search results, a “title” instead of “topic” search strategy was used (14–16). The searching terms were “lateral epicondylitis”, “tennis elbow”, “elbow, tennis”, “elbows, tennis”, “epicondylitis, lateral”, “epicondylitis, lateral humeral”, “humeral epicondylitis, lateral”, and “lateral humeral epicondylitis” with the use of the “OR” operator.

### 2.2 Article selection criteria

**Figure 1:**
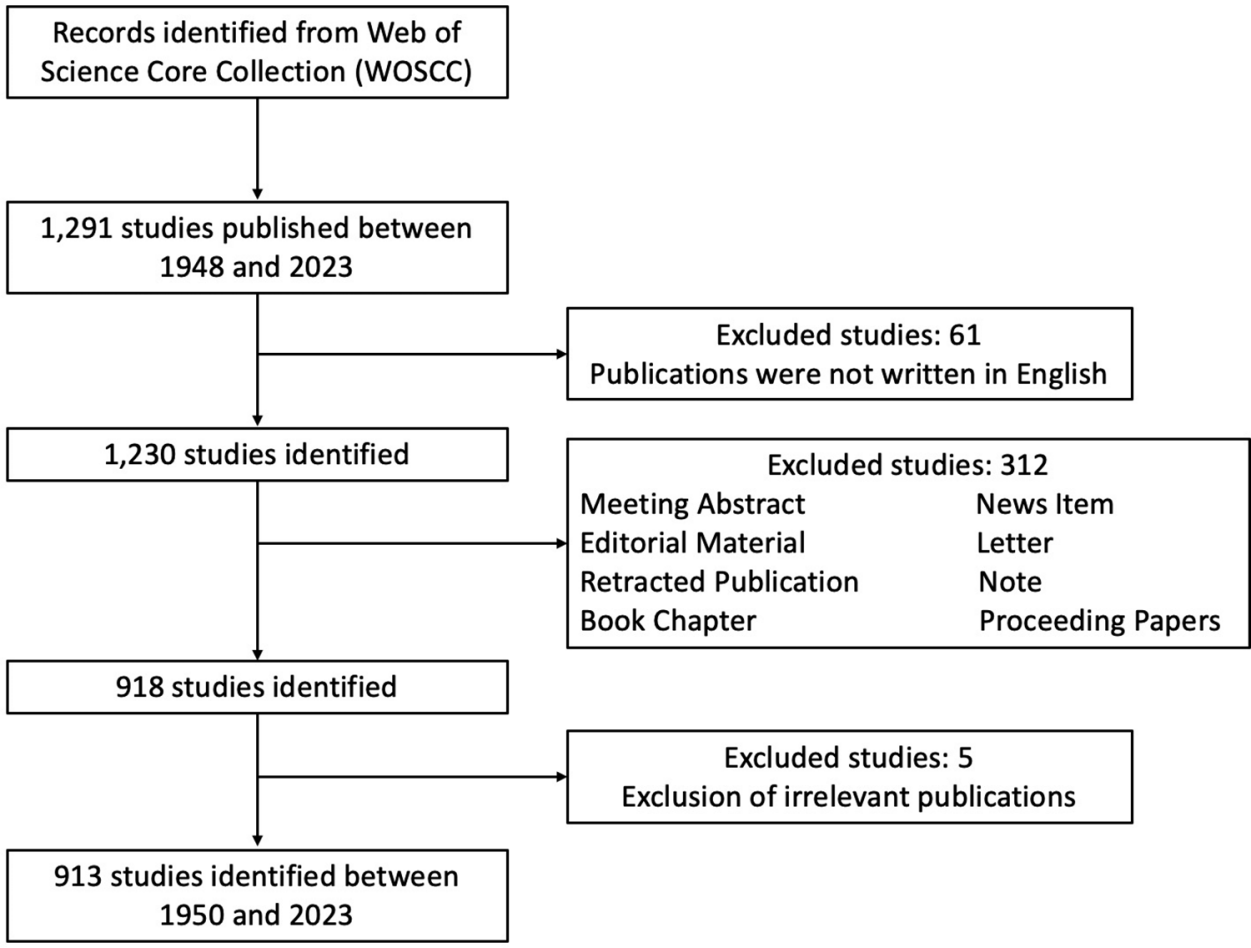
Flow diagram of screening process concerning lateral epicondylitis research.

Two independent authors screened the abstracts or full texts of articles pertaining to LE. A third reviewer was consulted to achieve consensus when there were disagreements between these two reviewers. The publication type was refined as article or review. Meeting abstracts, letters, notes, proceeding papers, book chapter, or editorial materials were excluded. References of the eligible articles were checked for any additional related articles. Only articles written in English were included in our study. The detailed selection criteria and inclusion procedure of this study were shown in Figure 1.

### 2.3 Data extraction

Data of publications extracted from the WOSCC included: number of citations, authors' name, article title, organization, country, journal, year of publication, keywords, topic of publication, and H-index. The H-index, also known as the H-factor, is a new approach to assess academic achievement, a researcher's H-index is when she or he has at most H papers that are referred to at least H times. The higher a researcher's H-score, the stronger the impact of her or his scientific work (17).

### 2.4 Statistical analysis

Microsoft Excel 2020 and GraphPad Prism v. 7.0 (GraphPad, La Jolla, CA, USA) were used to analyze the bibliometric characteristics. VOSviewer 1.6.16 (Leiden University, Leiden, Netherlands) was applied to analyze and visualize the co-citation of authors and references, also the co-occurrence of keywords and hot topics. Here, the line thickness between the colored points indicates the total link strength, while the size of colored points represents the number of publications in bibliographic coupling. Different colors stand for different clusters in the network maps.

## 3 Results

### 3.1 Overview of publications on LE

A total of 913 publications were retrieved from WOSCC based on our inclusion criteria. The total number of citations was 27,866 (18,981 without self-citations). The average number of citations was 30.52 and the H-index of all selected articles concerning LE was 78.

### 3.2 Year of publication

**Figure 2:**
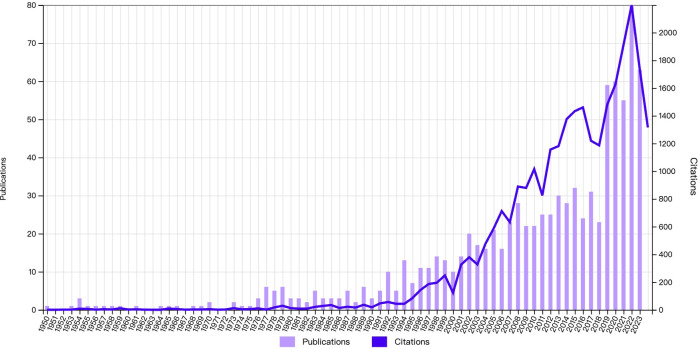
Publication and citation trends on lateral epicondylitis from 1950 to 2023.

Publication years of studies on LE ranged from 1950 to 2023 with the number of publications and citations per year both generally increasing (Figure 2). The most productive years were 2022 (*n* = 80), followed by 2023 (*n* = 63) and 2020 (*n* = 60).

### 3.3 Topics of study

**Figure 3:**
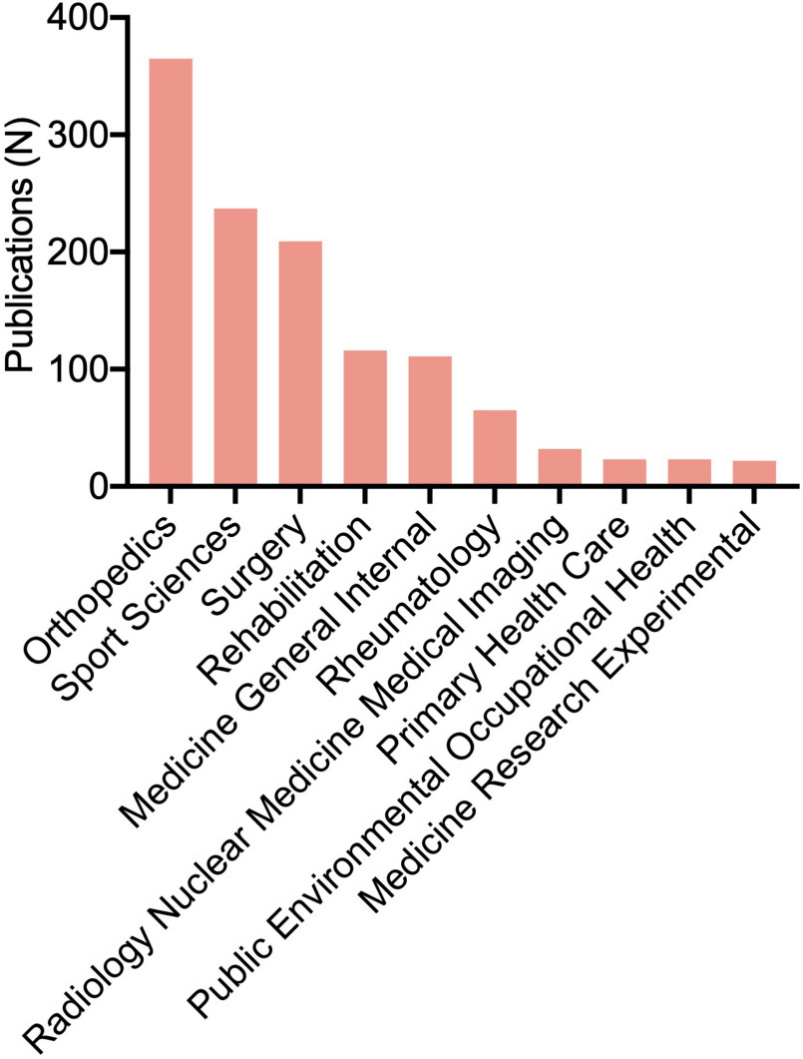
The most prominent research topics on lateral epicondylitis.

In all included articles, “Orthopedics” was the dominant topic (*n* = 365, 40.0%), followed by “Sport Sciences” (*n* = 237, 26.0%) and “Surgery” (*n* = 209, 22.9%) (Figure 3).

### 3.4 Distribution of publication journals

The *Journal of Shoulder and Elbow Surgery* contributed the most publications (IF = 2.9, *n* = 39, 4.3%), followed by *The American Journal of Sports Medicine* (IF = 4.2, *n* = 33, 3.6%) and *Journal of Hand Surgery (American Volume)* (IF = 2.1, *n* = 19, 2.1%) (Table 1).

**Table 1:** Top 10 productive journals of publications on lateral epicondylitis.

Journal	Publications (*N*)	Total citations (*N*)	H-index	Impact factor
Journal of Shoulder and Elbow Surgery	39	911	16	2.9
American Journal of Sports Medicine	33	2,931	26	4.2
Journal of Hand Surgery-American Volume	19	765	13	2.1
Arthroscopy-the Journal of Arthroscopic and Related Surgery	18	496	12	4.4
British Journal of Sports Medicine	17	677	13	11.6
Clinical Orthopaedics and Related Research	17	596	14	4.2
BMC Musculoskeletal Disorders	16	297	9	2.2
Archives of Orthopaedic and Trauma Surgery	13	358	11	2.0
Journal of Bone and Joint Surgery-American Volume	12	2,040	12	4.4
Physician and Sports Medicine	12	90	4	1.9

*N*, number.

### 3.5 Countries and organizations of publications

The United States contributed the most articles (*n* = 201, 22.0%). The United Kingdom ranked the second (*n* = 94, 10.3%), followed by Turkey (*n* = 85, 9.3%) (Table 2). The top three productive organizations were University of Amsterdam (*n* = 31, 3.4%), Vrije Universiteit Amsterdam (*n* = 15, 1.6%) and Keele University (*n* = 13, 1.4%) (Table 3).

**Table 2:** Top 10 contributing countries of publications.

Country	Publications (*N*)	Total citations (*N*)	H-index
USA	201	7,573	48
UK	94	3,739	37
Turkey	85	915	17
South Korea	45	360	12
China	40	529	13
Netherlands	40	3,111	26
Australia	36	1,469	21
Canada	35	1,176	18
Germany	35	1,424	21
Sweden	33	1,184	20

*N*, number.

**Table 3:** Top 10 productive organizations for the research.

Organization	Publications (*N*)	Total citations (*N*)	H-index
University of Amsterdam	31	2,479	22
Vrije Universiteit Amsterdam	15	1,316	11
Keele University	13	545	11
Egyptian Knowledge Bank EKB	12	91	3
Umea University	12	582	9
Chang Gung Memorial Hospital	11	132	4
Hosp Special Surg	11	257	5
Mayo Clinic	10	514	7
University of California System	10	326	8
Karolinska Institutet	9	342	7

*N*, number.

### 3.6 Bibliometric maps of co-citations

**Figure 4:**
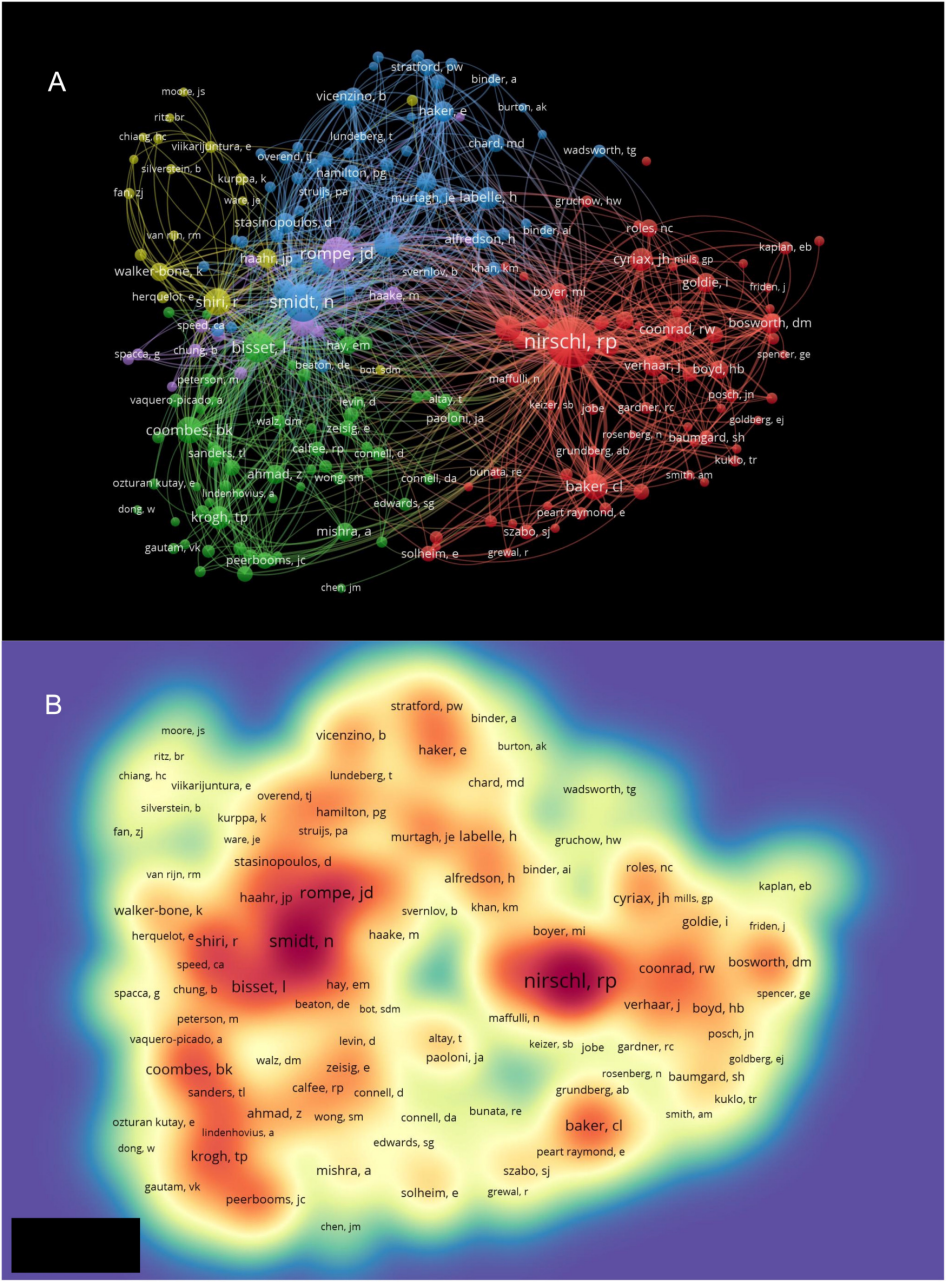
**(A)** Mapping on co-cited references of studies related to lateral epicondylitis. The size of a point indicates the citation number of the publication. The line between two points represents that both publications had been cited in one paper. The length of a line represents the closeness of the two publications; the link is closer; the length of the line is shorter. **(B)** Mapping on density visualization of co-cited references. Different colors represent different citation frequency. Purple represents few times; green represents average times and red represents many times. Items in one red circle linked closer with each other than that in other color areas.

Co-citation analysis of references is based on inferring the similarity between two references by the number of co-cited times, which is one of the most important aspects in bibliometric analysis. The minimum number of citations of a cited reference was set as 20 of the 9,521 cited references, 188 met the threshold and were subjected to analysis. VOSviewer was applied to analyze and visualize the total strength of co-citation links among cited references. The first cluster included 65 publications and mainly focused on the surgical treatment of LE. The second cluster incorporated 56 publications and focused on histological and immunohistochemical aspects associated with LE. The third cluster contained 54 publications and focused on injection therapies. The fourth cluster encompassed 13 publications and focused on the extracorporeal shockwave therapy (Figure 4).

**Figure 5:**
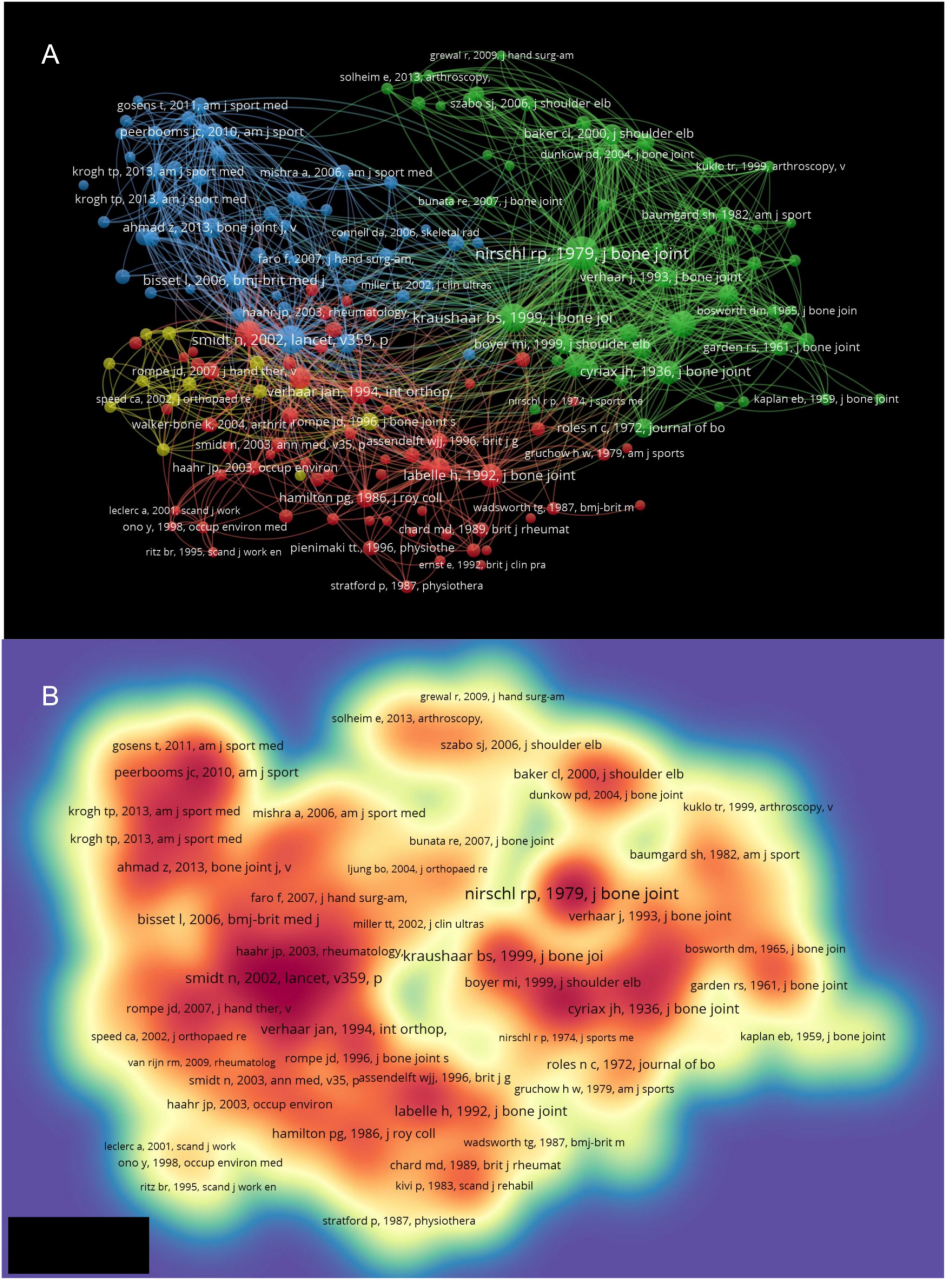
**(A)** Mapping on co-cited authors of studies related to lateral epicondylitis. The bigger size of a point represents more citation number of the author. The line between two points means that both authors had been cited in one publication. The length of a line represents the closeness of the two authors; shorter line means the link is closer. **(B)** Mapping on density visualization of co-cited authors. Different colors represent different citation frequency. Purple represents few times; green represents average times and red represents many times. Items in one red circle linked closer with each other than that in other color areas.

Co-citation analysis of authors was also performed by VOSviewer. The minimum number of citations of an author was set as 20. Of 6,957 authors, 221 met the threshold and were analyzed. Nirschl RP had the highest number of citations of 566 and owned the strongest total link strength of 9,364 (Figure 5). In the present study, the most robust total link strength was observed among authors who were considered active collaborators. Nirschl RP, Rompe JD, and Smidt N were identified as three leading authors with a wide-ranging cooperative network. In accordance with the established criteria, all three authors were identified as pioneers or technology innovators in the field of LE.

### 3.7 Bibliometric maps of co-occurrence

**Figure 6:**
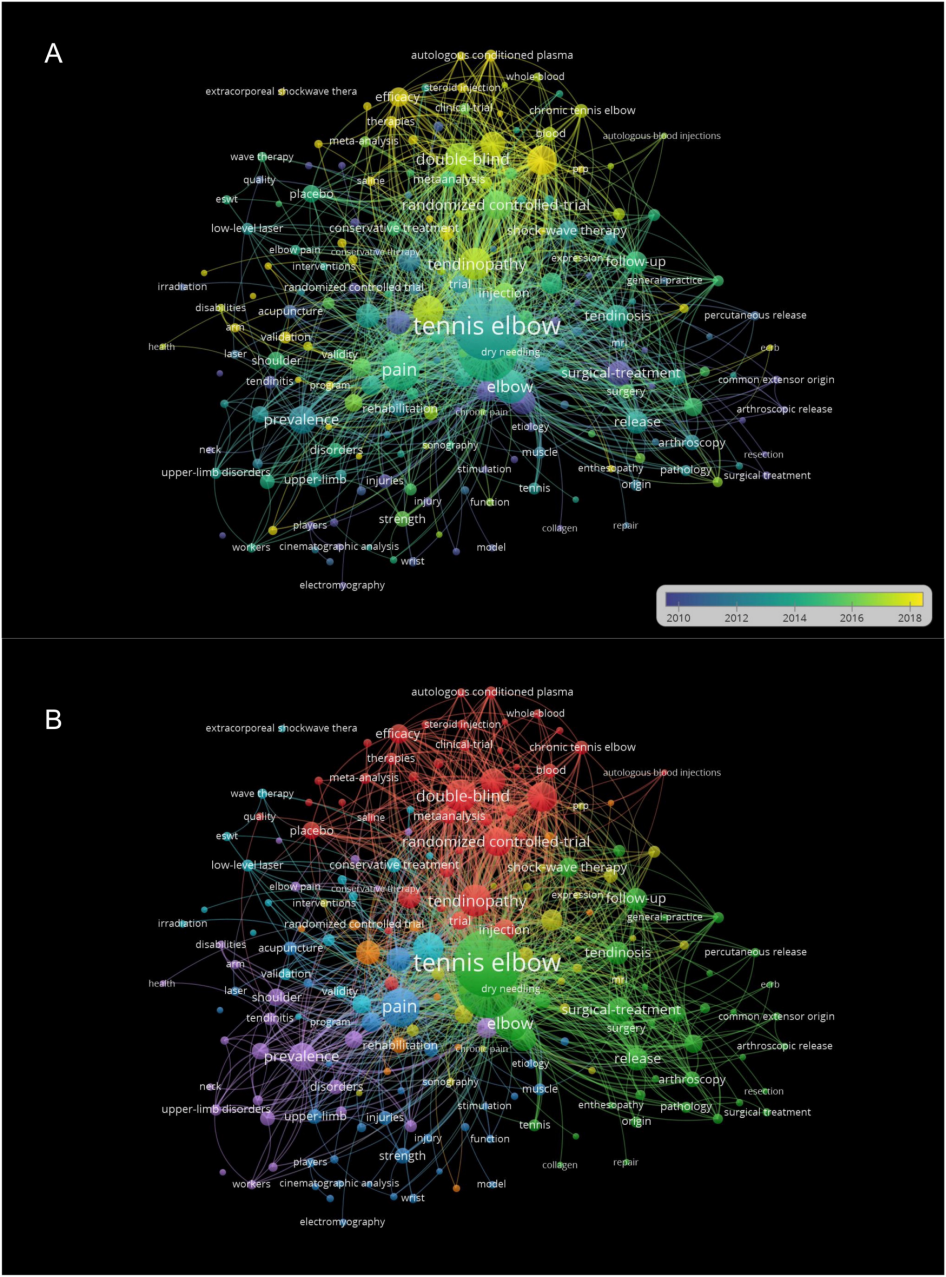
**(A)** Visualization of the keyword appearing time. Keywords in yellow appeared later than that in blue. **(B)** Mapping on co-occurrence of keywords related to lateral epicondylitis. The size of a point indicates the frequency of the keywords. The line between two points represents that both keywords occurred in one publication.

The minimum number of occurrences of a keyword was set as 5 of the 1,738 keywords, and 201 met the threshold. The top three keywords were “tennis elbow”, “lateral epicondylitis”, and “pain”, with 473, 380, and 162 occurrences, respectively. The latest keywords “platelet rich plasma”, “autologous conditioned plasma”, and “extracorporeal shockwave therapy” mainly appeared since 2018 (Figure 6A).

The keywords were classified into seven clusters. Cluster 1 included 43 keywords, which were mainly focused the surgical treatment. Cluster 2 consisted of 40 keywords that mainly discussed anatomy. Cluster 3 comprised of 35 keywords that were mainly related to the grip strength. Cluster 4 contained 26 keywords that were mainly concentrated on injection therapy. Cluster 5 incorporated 25 keywords that were mainly focused on prevalence. Cluster 6 consisted of 19 keywords that were mainly focused on extracorporeal shockwave therapy. Cluster 7 involved 13 keywords that were mainly focused on prognosis and rehabilitation of LE (Figure 6B).

## 4 Discussion

LE affects 1% to 3% of the population and is one of the most diagnosed musculoskeletal conditions of the elbow (3, 4, 18). Although a large number of treatment options have been developed for LE, there is still a controversy regarding the optimal treatment (3, 19). Therefore, it is important to analyze all publications on LE for grabbing a more comprehensive understanding of current global research status and future hot spots in this field, which may provide evidence for various treatment options. We evaluated the present research activity and future trends of LE thoroughly via bibliometric analysis and a total of 913 publications from the WOSCC database were included. Based on our analysis, the annual publications from 1950 to 2000 were relatively rare, indicating the research on LE was not enough. While the number of publications and total citations have grown fast since 2000, suggesting an increasing interest among scholars in relation to the biological and clinical outcomes in LE research. The total citations of these 913 publications reached 27,866, which was higher than that for robotic arthroplasty area (*n* = 27,461) (10). The discrepancy may be attributable to the size of the professional community and its respective interests (20).

The *Journal of Shoulder and Elbow Surgery*, *The American Journal of Sports Medicine* and *Journal of Hand Surgery American Volume* were the top three productive journals, suggesting that more research on this area will be published in these journals in the future. Although the *Journal of Shoulder and Elbow Surgery* did not own the highest impact factor, due to it was one of the few journals that concentrate on shoulder and elbow surgery specially, it contributed the highest number of publications. Researchers that are focused on LE may pay more attention to these journals. The United States ranked the first in the number of total publications, citations, and H-index, which indicated the United States dominated studies of LE. This was also seen in other research topics (21–23). The possible reason is the superior impact of science and technology budgets of this country and the financial support of organizations (24, 25).

The keyword co-occurrence analysis can help researchers to obtain hot topics on LE accurately. We found that “platelet rich plasma”, “autologous conditioned plasma”, and “extracorporeal shockwave therapy” may be new hotspots. Corticosteroid injections are considered as a frequently used treatment modality for LE historically, but an increasing number of studies suggest their lack of efficacy (26–28). Instead, platelet rich plasma and autologous conditioned plasma injection therapy have been more used to treat LE recently, which have been proven as effectively (4, 29, 30). Platelet rich plasma is abundant in platelets, growth factors, and fibrin, which promotes the proliferation of tendon cells and angiogenesis, also has an anti-inflammatory and tissue-repairing potential (31). Owning to the non-invasive nature and little to no side effects or adverse events on extracorporeal shockwave therapy, it is preferred by many patients and clinicians as a probably alternative to reduce pain and medical expenses associated with more invasive therapy (32). Additionally, the efficacy of this therapy has been supported by a growing number of clinical studies (33, 34). However, there still exists contradictory on the effectiveness of extracorporeal shockwave therapy for the management of LE (35). The effectiveness of extracorporeal shockwave therapy deserves further research, and more focus is required to instruct clinical practice.

When making the co-citation analysis of references, we found an article entitled “Tennis elbow. The surgical treatment of lateral epicondylitis” in the first cluster was the most cited article with the total link strength of 2,728, which was published in the *Journal of bone and joint surgery (American volume)* in 1979 by Nirschl RP et al. (36). This publication indicated the consistent lesion of 88 lateral tennis elbow cases was immature fibroblastic and vascular infiltration from the origin of the extensor carpi radialis brevis. Then, a specific surgical technique was applied, including exposure of the extensor carpi radialis brevis, excision of the identified lesion, and repair, which presented an overall improvement rate of 97.7%. The significance of this classical study was to reveal that surgical intervention can be an option for patients with recalcitrant pain and disability which have failed appropriate nonoperative treatment.

In the second cluster, Kraushaar BS et al. published an article titled “Tendinosis of the Elbow (Tennis Elbow). Clinical Features and Findings of Histological, Immunohistochemical, and Electron Microscopy Studies” in *Journal of Bone and Joint Surgery (American Volume)* in 1999 with a total link strength of 2,052 (37). They confirmed that inflammatory cells were conspicuously absent, along with mesenchymal differentiation and metaplasia were primary cellular events of LE. The histological and immunohistochemical research might generate fresh theories about the pain management by improving currently used interventions and developing new techniques.

In the third cluster, Smidt N's publication titled “Corticosteroid injections, physiotherapy, or a wait-and-see policy for lateral epicondylitis: a randomised controlled trial”, which was published in the *Lancet* in 2002, owned the total link strength of 2,154 (38). This study suggested that although corticosteroid injection therapy can relieve acute symptoms quickly, they were unsuitable for long-term cases. Recent studies reported platelet rich plasma and autologous conditioned plasma injection therapy have been developed and utilized to treat LE widely (29, 30). However, there still exists the questionable evidence of its efficacy for the treatment (39). In general, injection therapy has gained more popularity and interests in recent years for the management.

In the fourth cluster, *American Journal of Sports Medicine* published an article entitled “Repetitive low-energy shock wave treatment for chronic lateral epicondylitis in tennis players” in 2004, which was written by Rompe JD et al., with a total link strength of 464 (40). This clinical trial study was designed based on the conflicting evidence regarding extracorporeal shock wave treatment for chronic LE, which demonstrated that repetitive low-energy extracorporeal shock wave treatment was superior to repetitive placebo extra-corporeal shock wave treatment.

Notably, extracorporeal shockwave therapy and injection therapy remained high interests in both co-occurrence and co-citation analysis. Researchers who are interested in LE should pay more attention to these topics. The prevailing approach for addressing LE in clinical cases is conservative management, which involves the use of oral corticosteroids, bracing, and activity restriction (41). However, there is a paucity of studies that have examined the effectiveness of such treatment modalities (41, 42). The integration of radiological diagnosis with clinical assessment has the potential to facilitate the development of management that are tailored to the individual needs of patients with LE (43). Recently, as a cost effective and short time-consuming method to display soft tissue lesions, ultrasound (US) imaging in diagnosing and managing LE has become more and more essential due to it is beneficial to identify different types of lateral elbow tendinopathy (e.g., tendinosis, tendon hyperemia, and tendon delamination) (44). Moreover, it can be rendered more sensitive to conservative, interventional, and surgical approaches, which in turn, facilitate precise targeting and guidance during the onward procedure. Consequently, the integration of US imaging with clinical findings can facilitate a more precise characterization of the underlying pain generator, thereby enabling enhanced management of patients with LE (45).

## 5 Limitations

There are several limitations in the present study. First, although the WOSCC is one of the most comprehensive and widely used database, it might not consist of specific publications that appeared in other sources such as Google Scholar, Scopus or PubMed, which might cause bias probably. Proposing alternative databases such as multi-database retrieval may enhance the rigor of the study. Second, only specific publications (English language, type of article and review) were included in our study, which may have led us to overlook high-quality literature in other languages and resulted in biased results. Third, H-index and the total number of citations are affected by time. Recent studies may have relatively low H-index values and cited times, resulting in difference between research results and actual situation. Lastly, we sought to identify only relevant studies in this field, therefore the “title” rather than “topic” searching strategy was applied. This might miss some, but most likely an insignificant number of relevant publications. Despite these limitations, our study provides value insights into the research activity and development on LE. Meanwhile, the optimal treatment for LE is still needed further research and more clinical studies with high quality (e.g., prospective studies and randomized controlled trials) are required in the future.

## 6 Conclusions

In conclusion, this bibliometric study indicates there is a growing trend in the number of publications on LE. The United States dominated studies and the *Journal of Shoulder and Elbow Surgery* was the most productive journal. The primary management strategy for LE in most clinical situations is conservative management, which includes the administration of oral corticosteroids, the use of a bracing apparatus, and the implementation of activity restriction. While “Platelet rich plasma”, “autologous conditioned plasma”, and “extracorporeal shockwave therapy” may become new interests in LE research. High-quality studies focused on the effectiveness of clinical trials and radiological diagnosis are still required. The present study can be used as a feasible solution for LE research, which will benefit patients and related professionals in practice.

## Data Availability

The original contributions presented in the study are included in the article/Supplementary Material, further inquiries can be directed to the corresponding author.
